# Comparative Evaluation of Machine Learning-Based Radiomics and Deep Learning for Breast Lesion Classification in Mammography

**DOI:** 10.3390/diagnostics15080953

**Published:** 2025-04-09

**Authors:** Alessandro Stefano, Fabiano Bini, Eleonora Giovagnoli, Mariangela Dimarco, Nicolò Lauciello, Daniela Narbonese, Giovanni Pasini, Franco Marinozzi, Giorgio Russo, Ildebrando D’Angelo

**Affiliations:** 1 Institute of Bioimaging and Complex Biological Systems, National Research Council (IBSBC-CNR), Contrada, Pietrapollastra-Pisciotto, 90015 Cefalù, Italy; alessandro.stefano@cnr.it (A.S.); nicolo.lauciello@unipa.it (N.L.); giovanni.pasini@uniroma1.it (G.P.); giorgio-russo@cnr.it (G.R.); 2 Department of Mechanical and Aerospace Engineering, Sapienza University of Rome, Eudossiana 18, 00184 Rome, Italy; giovagnoli.1918945@studenti.uniroma1.it (E.G.); franco.marinozzi@uniroma1.it (F.M.); 3 Department of Radiology, Fondazione Istituto “G. Giglio”, 90015 Cefalù, Italy; maridimarco33@gmail.com (M.D.); daniela.narbonese@studenti.unipd.it (D.N.); ildebrando.dangelo@hsrgiglio.it (I.D.); 4 Department of Earth and Marine Sciences, University of Palermo, Via Archirafi 22, 90123 Palermo, Italy

**Keywords:** mammography, radiomics, deep learning, automated diagnostic systems, breast lesion classification

## Abstract

**Background:** Breast cancer is the second leading cause of cancer-related mortality among women, accounting for 12% of cases. Early diagnosis, based on the identification of radiological features, such as masses and microcalcifications in mammograms, is crucial for reducing mortality rates. However, manual interpretation by radiologists is complex and subject to variability, emphasizing the need for automated diagnostic tools to enhance accuracy and efficiency. This study compares a radiomics workflow based on machine learning (ML) with a deep learning (DL) approach for classifying breast lesions as benign or malignant. **Methods**: matRadiomics was used to extract radiomics features from mammographic images of 1219 patients from the CBIS-DDSM public database, including 581 cases of microcalcifications and 638 of masses. Among the ML models, a linear discriminant analysis (LDA) demonstrated the best performance for both lesion types. External validation was conducted on a private dataset of 222 images to evaluate generalizability to an independent cohort. Additionally, a deep learning approach based on the EfficientNetB6 model was employed for comparison. **Results**: The LDA model achieved a mean validation AUC of 68.28% for microcalcifications and 61.53% for masses. In the external validation, AUC values of 66.9% and 61.5% were obtained, respectively. In contrast, the EfficientNetB6 model demonstrated superior performance, achieving an AUC of 81.52% for microcalcifications and 76.24% for masses, highlighting the potential of DL for improved diagnostic accuracy. **Conclusions**: This study underscores the limitations of ML-based radiomics in breast cancer diagnosis. Deep learning proves to be a more effective approach, offering enhanced accuracy and supporting clinicians in improving patient management.

## 1. Introduction

Breast cancer is the most common malignancy among women worldwide, second only to lung cancer, affecting approximately 10–12% of the female population and causing around 500,000 deaths annually [1]. The highest incidence occurs in women aged 40–49 years (41%), with a lower prevalence in those over 71 (21%). Risk factors include genetic predisposition (e.g., BRCA1/BRCA2 mutations), lifestyle, prolonged hormone therapy, therapeutic radiation, and benign proliferative breast diseases [2]. Early stages are often asymptomatic, but initial signs may include hard lesions, breast shape alterations, nipple changes, or unexplained weight loss. Advancements in therapeutic strategies and screening programs have progressively reduced mortality rates. The World Health Organization (WHO) revised its 2003 classification to improve lesion characterization, distinguishing categories such as intraductal proliferative lesions, papillary lesions, mesenchymal and fibroepithelial tumors, and rarer malignancies, like lymphomas and metastases [3,4]. Additionally, abnormalities, such as masses and microcalcifications, while not strictly histological, are key mammographic indicators of breast cancer risk [5]. Accurate diagnosis begins with medical history, clinical examination, and imaging techniques. Mammography remains the gold standard for early detection, particularly in asymptomatic cases, but tissue overlap can hinder interpretation, leading to false positives or negatives [6,7,8]. To address these limitations, tomosynthesis provides a near 3D breast reconstruction, reducing tissue superimposition and improving lesion detection [9,10]. Ultrasound complements mammography by distinguishing solid from cystic formations, particularly in dense breast tissue, where mammography is less effective [11,12]. MRI further enhances sensitivity (up to 92%) but has lower specificity (~60%) due to challenges in lesion characterization [13,14]. The Breast Imaging Reporting and Data System (BI-RADS) ensures standardized reporting, aiding risk assessment and clinical decision-making [15]. Computer-aided diagnosis (CAD) systems refine mass classification, improving diagnostic precision [16]. Radiomics is gaining prominence for its ability to extract high-dimensional quantitative features (e.g., shape, texture, intensity) from medical images, correlating them with clinical outcomes [17,18,19,20]. The radiomics workflow includes image acquisition, preprocessing, tumor segmentation, feature extraction, and machine learning (ML)-based model development. Deep learning (DL) advancements have expanded radiomics applications, enabling predictive modeling for cancer prognosis and treatment response [21,22,23,24]. DL models have shown remarkable ability to automatically extract hierarchical features from raw imaging data, often outperforming traditional ML workflows that rely on handcrafted radiomics features [25,26,27,28]. Specifically, convolutional neural networks (CNNs) have demonstrated high accuracy in cancer risk assessment and prognosis prediction [29,30,31]. Zhang et al. [32] highlighted the value of attention mechanisms and ensemble learning strategies in enhancing model robustness and generalization across multicenter datasets. Among CNNs, EfficientNet [33] employs a compound scaling method, which simultaneously scales the depth, width, and input resolution in a balanced manner. This design enables higher accuracy with fewer parameters and lower computational costs than conventional architectures, such as ResNet [34], DenseNet [35], DeepLabV3+ [36], or Inception [37]. EfficientNetB6 offers an optimal trade-off between model capacity and computational efficiency, making it suitable for medical imaging tasks that involve subtle visual differences, such as distinguishing benign from malignant breast lesions. Secondly, several recent studies have demonstrated the superior performance of EfficientNet variants in medical image classification, including mammography, due to their ability to capture fine-grained textural and morphological features. For instance, Tan and Le [33] showed that EfficientNet models outperform deeper conventional CNNs on benchmark datasets, and more recent applications have validated their use in radiology workflows, particularly in breast cancer imaging [38,39].

This study aims to directly compare a radiomics-based ML pipeline (i.e., matRadiomics [40,41]) with a DL approach (i.e., EfficientNet) in classifying breast lesions as benign or malignant using the CBIS-DDSM (Curated Breast Imaging Subset of Digital Database for Screening Mammography) [42] public mammography dataset. Specifically, matRadiomics was integrated with preprocessing techniques to improve the mammographic image quality. Subsequently, ML classifiers were tested for their ability to classify breast masses and microcalcifications. A private dataset was used for external validation, emphasizing the importance of model generalization in breast lesion classification. By juxtaposing ML-based radiomics with DL, this research highlights the potential of advanced methodologies to improve the accuracy and reliability of diagnosis. By addressing key challenges, such as methodological standardization and clinical applicability [43], this work contributes to the evolving role of image processing in automated breast cancer diagnosis.

## 2. Materials and Methods

This section outlines the tools and methodologies used for the radiomics and DL analyses. The CBIS-DDSM database [42], containing mammographic images and lesion segmentation masks, was used. The analysis used matRadiomics [40,41] with the integration of preprocessing techniques implemented specifically for this type of image. Furthermore, the EfficientNetB6 convolutional neural network was used to explore the potential of DL and compare it with traditional ML methods for lesion classification.

### 2.1. Datasets

#### 2.1.1. Dataset for Internal Validation

The CBIS-DDSM database comprises mammographic images available for public download from the Cancer Imaging Archive (TCIA), a repository of medical images of cancer. This dataset divided the images into microcalcifications (1872 images) and masses (1696 images), representing 753 and 891 patients, respectively. Among these 3568 images, 2111 cases are benign, while 1457 are malignant. Each patient has multiple IDs corresponding to scans from different projections (e.g., craniocaudal (CC) or mediolateral oblique (MLO)) and specific anomalies, identified by unique codes. For each patient, there are at least two series of images: a full mammogram and additional images highlighting the lesion, including a segmented region of interest (ROI) and a cropped image of the affected area. Some patients exhibit both types of anomalies. All images are in a DICOM (Digital Imaging and Communications in Medicine) format with digital breast tomosynthesis extensions. The database includes comprehensive metadata in CSV files, detailing breast density (based on the BI-RADS system), breast side (left or right), image view (CC or MLO), anomaly identifiers, anomaly type (mass or calcification), and specific characteristics, such as mass shape, calcification type, margins, distribution, BI-RADS assessment, pathology (benign or malignant), and anomaly subtlety (graded from 1 to 5). These details offer a rich dataset for exploring breast cancer imaging [42].

#### 2.1.2. Dataset for External Validation

The external validation of the model was conducted using a database provided by the Fondazione Istituto “G. Giglio” of Cefalù” that collected all patient cases available over the study timeframe. Therefore, this dataset was not specifically designed to be balanced or matched to the internal training/validation dataset in terms of patient-to-image ratio or class distribution. This cohort serves to assess the generalizability of the proposed workflow in an independent and real-world clinical setting. This dataset consists of 222 images from 180 patients, including 149 cases with calcifications and 73 with masses. This retrospective study was approved by the local ethical review board.

### 2.2. Toolbox for Radiomics Analysis: matRadiomics

matRadiomics is a user-friendly, Image Biomarker Standardization Initiative (IBSI)-compliant framework designed to optimize and simplify the radiomics workflow in both clinical and preclinical contexts. Built on MATLAB (v. r2021b) and Python (v. 3.7.0), it integrates several powerful tools for medical image analysis, including the PyRadiomics library for feature extraction [44], ComBat for data harmonization [45], and various ML algorithms for predictive modeling. The platform supports the processing of medical images from different modalities, such as Positron Emission Tomography (PET) [46,47], CT (Computed Tomography) [48], and MRI (Magnetic Resonance Imaging) [49], and facilitates lesion classification and differentiation, including benign vs. malignant lesions. Key functionalities of matRadiomics include the ability to create and import radiomics studies, perform target segmentation, and extract radiomics features. To enhance image quality before feature extraction, we incorporated image preprocessing algorithms, like Contrast Limited Adaptive Histogram Equalization (CLAHE) and median filter.

### 2.3. Preprocessing

Two preprocessing techniques were applied to improve mammographic image quality and optimize lesion classification: CLAHE and median filter. These methods enhance image quality, leading to more accurate feature extraction and predictive modeling [50,51]:

- CLAHE increases contrast in low-contrast mammographic images while minimizing noise amplification.
- The median filter reduces noise while preserving critical image structures, outperforming traditional average filters [52].

Specifically, CLAHE is an advanced histogram equalization technique designed to enhance local contrast while preventing excessive noise amplification and artifacts in high-intensity regions. Unlike traditional histogram equalization, which operates on the entire image, CLAHE divides the image into small, non-overlapping tiles and computes a local histogram for each tile. The histogram is then clipped at a predefined limit to avoid the over-amplification of certain intensity levels. After clipping, the modified histogram is normalized and used to remap the pixel intensities, enhancing local contrast.

In summary, CLAHE can achieve the following:

- Improve the visibility of fine details and textures, making key diagnostic patterns more evident, which helps both traditional radiomics and DL models extract more informative features.
- Reduce the impact of lighting or acquisition variations across images, leading to more consistent input data for the model.
- Improve the discrimination performance because better feature representation often translates into better performance, especially in distinguishing small lesions or subtle abnormalities.

In addition, taking into account the characteristics of the images and the noise that often degrades visual quality and obscures critical details, among the denoising techniques emerges the median filter, which is particularly effective for removing impulsive noise while preserving edges and fine details. Unlike linear filters, which average pixel values and may blur edges, the median filter operates non-linearly, replacing each pixel with the median value of its surrounding neighborhood. To formalize this process, consider a window of size N × N (e.g., 3 × 3) that slides across the image and $X=\left\{x_{1},x_{2}\ldots x_{n}\right\}$ and the pixel values within that window. The median filter, or median (*X*), replaces the value of pixel $f^{\prime }\left(x_{i}\right)$ with the median of its neighbors in the window as follows:

(1) $$f^{\prime }\left(x_{i}\right)=median(X)$$

By selecting the median rather than the mean, this method effectively removes outlier values caused by noise, preventing excessive blurring and preserving the structural integrity of the image. Together, these preprocessing techniques enhance the image contrast and reduce noise, highlighting areas of interest while maintaining the integrity of the image’s structural details [53].

### 2.4. Radiomics Features

Radiomics features are quantitative attributes extracted from medical images, providing critical insights into tissue properties and lesion morphology. These features are derived through advanced algorithms [54,55] and are typically classified into three categories:

1. First-order features describe the intensity distribution within the region of interest (ROI), including metrics such as mean, standard deviation, skewness, and kurtosis.
2. Second-order features analyze texture by assessing the spatial relationships between voxel intensities, capturing patterns that reflect tissue heterogeneity.
3. Shape features define the geometry and morphology of the ROI, including volume, surface area, and sphericity. These are particularly useful for distinguishing lesions, as benign ones tend to be more regular, while malignant ones often exhibit irregular shapes.

#### 2.4.1. Feature Extraction

Three image transformations were applied—original, wavelet, and LoG (Laplacian of Gaussian)—to capture a broader range of structural details. While original images preserve raw data, wavelet transformations decompose images into low- and high-frequency components, enhancing fine textures [56], and LoG filtering improves edge detection and highlights local intensity variations, aiding in the identification of subtle patterns. For wavelet decomposition, the Haar method was chosen. For LoG, sigma equal to 1 was chosen. To balance texture preservation and noise reduction, discretization was set to 64 bins, a value supported by previous studies as an effective trade-off [57]. However, in the preliminary stages of our study, we performed comparative tests using two different bin sizes (i.e., 32 and 128) to assess their potential impact on the model performance. The results showed only marginal differences in classification metrics, thus not affecting the aim of our study; the value of 64 was ultimately chosen because it was supported by a bibliographic reference [57].

#### 2.4.2. Feature Selection

Feature selection is a crucial step in the radiomics pipeline, reducing redundancy and non-informative features to enhance model performance, lower computational costs, and improve generalization [58,59,60]. matRadiomics offers four different selection methods [41]: *t*-test, relieff, Least Absolute Shrinkage and Selection Operator (LASSO), and a descriptive-inferential sequential approach based on point biserial correlation (PBC). Among the available techniques, the PBC-based method showed the best results. This method was implemented by our group, as described in detail in [61]. Briefly, the PBC ranks features based on their correlation with the binary target variable (e.g., benign vs. malignant tumors). Consequently, for each feature, the index between the features and the dichotomous outcome is calculated, sorting the features in a decreasing PBC order. Then, a cycle starts to add one column at a time, performing a logistic regression analysis by comparing the *p*-value of each iteration and stopping in the case of a growing *p*-value. This approach is particularly effective in high-dimensional datasets with non-standard distributions [62,63].

### 2.5. Machine Learning Predictive Models

The ML methods used in this work belong to the supervised learning category, in which there is a set of labeled training data (benign tumor labeled 1 and malignant tumor labeled 0) used to estimate or map the input data to the desired output. Based on matRadiomics, after the feature selection process, a linear discriminant analysis (LDA) and support vector machine (SVM) were used through k cross-validation.

#### 2.5.1. Linear Discriminant Analysis

LDA is a statistical ML technique primarily used for supervised classification. The goal of LDA is to project high-dimensional data onto a lower-dimensional space while maximizing the separation between classes. Specifically, LDA identifies a projection direction that maximizes the separation between class means while minimizing the variance within each class.

LDA relies on two key scatter matrices:

- Within-class scatter matrix ($S_{w}$): measures the variance within each class and should be minimized for optimal classification.
- Between-class scatter matrix ($S_{b}$): measures the variance between class means and should be maximized.

The optimal projection direction will be the one that maximizes the ratio between the $S_{b}$ and the $S_{w}$. It will be given by the following formula:

(2) $$w^{*}=S_{w}^{-1}(\mu_{1}-\mu_{2})$$

where $\mu_{1}$ and $\mu_{2}$ are the centroids of the various classes.

$S_{w}$ is found as follows:

(3) $$S_{w}=\sum_{i=1}^{C}\sum_{x\in classe\;i}(x-\mu_{i}){\left(x-\mu_{i}\right)}^{T}$$

The method is straightforward to implement and computationally efficient, making it a popular choice in many practical applications. By reducing the dimensionality of a dataset, LDA simplifies complex problems and helps in visualizing data in a two- or three-dimensional space. Additionally, the focus on maximizing the separation between classes ensures that LDA often provides robust performance for problems where the classes are well separated. LDA is particularly effective in cases where the number of features is high compared to the number of samples, as it reduces the feature space to the most discriminative dimensions. Its mathematical foundation also provides clear interpretability, allowing practitioners to understand how the model achieves its classification decisions [64].

#### 2.5.2. Support Vector Machine

SVM is a supervised learning algorithm primarily used for binary classification. Its objective is to identify a hyperplane that best separates the two classes by maximizing the margin, i.e., the minimum distance between the data points of each class and the hyperplane itself. This ensures that the classification is robust and less sensitive to small variations in the data [65].

A hyperplane can be described by the following equation:

(4) w∗x+b=0

where *w* is the normal vector to the hyperplane, *b* is the bias, and *x* represents the data points. To find the optimal hyperplane, the aim is to solve the following problem:

(5) $$\underset{w,b}{\min}\frac{1}{2}{\left|\left|w\right|\right|}^{2}$$

With the following constraint:

(6) $$y_{i}\left(w\cdot x_{i}+b\right)\geq 1\;\;\forall i$$

where w is the plane normal vector, b is the bias, and $y_{i}$ are the labels of the data (+1 or −1).

After finding the hyperplane, a new point *x* is classified with the following:

(7) $$f\left(x\right)=sign(w*x+b)$$

### 2.6. Performance Metrics

The final step in a radiomics analysis is assessing the trained model’s performance in classifying mammographic lesions. In this study, two metrics are examined: the Area Under the Curve (AUC) of the Receiver Operator Characteristic (ROC) curve and accuracy. These metrics are widely used in medical applications as they provide complementary insights into the classifier’s ability to distinguish between classes and make reliable predictions.

The true positive rate (TPR) and false positive rate (FPR), which serve as the axes of the ROC curve, are calculated from the following:

- True positives (TP): positive examples correctly classified as positive.
- True negatives (TN): negative examples correctly classified as negative.
- False positives (FP): negative examples incorrectly classified as positive.
- False negatives (FN): positive examples incorrectly classified as negative.

These two rates are then obtained as follows:

(8) $$TPR=\frac{TP}{P}=\frac{TP}{\left(TP+FN\right)}$$

Sensitivity, i.e., the TPR, which is the ratio between cases correctly classified as positive and all true positives, measures the ability of a model to correctly identify positives.

(9) $$FPR=\frac{FP}{N}=\frac{FP}{\left(FP+TN\right)}$$

The FPR represents the x-axis of the ROC space and is the ratio between the negative classes classified as positive and the true negatives.

These rates define the ROC curve, where TPR is plotted against FPR at various classification thresholds. An ideal classifier would achieve TPR = 1 (perfect sensitivity) and FPR = 0 (no false positives). To compare the performance of multiple classifiers and to summarize the performance of a classifier across all possible thresholds, the AUC is calculated, which is a numerical parameter that represents the classifier’s performance. AUC quantifies the overall ability of the classifier, and its value range is [0; 1]:

- An AUC of 1 indicates perfect classification, where the model distinguishes all positive from negative instances.
- An AUC of 0 suggests a completely inverted classifier.
- An AUC of 0.5 indicates random guessing, with no predictive power.

In addition, it is possible to calculate the Total Actual Positives (P) and Total Actual Negatives (N):

(10) P=TP+FN

(11) N=FP+TN

From these two metrics, accuracy can be derived as follows:

(12) $$ACC=\frac{TP+TN}{P+N}$$

Accuracy represents the proportion of correctly classified instances among all observations and provides a measure of the overall correctness, complementing the ROC curve and AUC. The choice of evaluation metrics depends on the clinical application. In this study, the primary goal is to determine whether a breast lesion is benign or malignant from a clinical decision support perspective. High sensitivity is crucial for the accurate identification of malignant cases, thereby minimizing false negatives. On the other hand, precision is especially important in situations with imbalanced datasets. Combining multiple metrics provides a more comprehensive evaluation of the model’s effectiveness [66,67,68].

### 2.7. Deep Radiomics

In addition to the conventional radiomics workflow, a deep radiomics approach was implemented using the EfficientNetB6. The selection of EfficientNetB6 was motivated by its unique compound scaling capability, which optimizes the network’s depth, width, and resolution simultaneously. This joint scaling strategy enables EfficientNet to achieve an optimal trade-off between performance and resource efficiency, reducing the number of parameters and computational operations (measured as FLOPS) compared to traditional architectures. The foundational architecture of EfficientNet, known as EfficientNetB0, incorporates Mobile Inverted Bottleneck Convolutions (MBConv), a design optimized for computational efficiency. Additionally, it integrates Squeeze-and-Excitation (SE) blocks, which enhance the model’s ability to capture relevant features by recalibrating channel-wise feature responses. These architectural innovations collectively improve both the accuracy and efficiency of the network. In this study, the EfficientNetB6 variant was chosen due to its balanced combination of computational complexity and predictive performance. Compared to other networks, EfficientNetB6 offers superior accuracy with a comparable number of parameters, making it particularly suitable for tasks that demand high performance without excessive computational cost. This balance is especially critical in medical imaging applications, where both precision and efficiency are paramount [33].

#### 2.7.1. Implementation of EfficientnetB6

The EfficientNetB6 network was pre-trained on the ImageNet dataset to leverage optimized weights, enhancing generalization on smaller datasets. The adaptation process included two phases: transfer learning and fine-tuning. The original classification head of the pre-trained model was replaced with a custom head that included fully connected layers. During this phase, only the new head was trained, while the weights of the convolutional layers were kept frozen to retain the knowledge acquired during the pre-training. In the fine-tuning phase, the entire model was refined. All weights, including those in the convolutional layers, were updated, allowing the model to adapt to the specific task while preserving the valuable features learned during the initial pre-training. In the case of mass classifications, the head was replaced with a Global Average Pooling operation, followed by a Dense Layer of 1024 neurons and a RELU activation function, a Dropout layer (percentage = 0.3), and a final Dense Layer of 1 neuron and a Sigmoid activation function, while, for calcification classifications, the head was replaced with a Global Average Pooling operation, followed by a Dense Layer of 256 neurons and a RELU activation function, a Dropout layer (percentage = 0.3), and a final Dense Layer of 1 neuron and a Sigmoid activation function. This is the configuration that maximizes the Area Under Curve metric on the validation set during training. For both calcification and mass classifications, the number of neurons tested in the Dense Layer ranged from 256 to 1024.

#### 2.7.2. Dataset Preparation for the Neural Network

The following steps were applied: (i) images were cropped to 512 × 512 pixels using a bounding box to isolate the lesion of interest. Padding was applied using image values rather than zeros to retain critical information; (ii) the same preprocessing techniques (CLAHE and median filtering) as in the classical radiomics workflow were used; (iii) to mitigate overfitting due to limited sample size, augmentation techniques were applied with random probabilities:

- Horizontal and vertical flips.
- Random translations and rotations (±20°).
- Brightness and contrast adjustments.
- Gaussian filtering and elastic deformations.

#### 2.7.3. Model Training Configuration

For mass classifications, the initial learning rate for the transfer phase was set to 1 × 10^−4^ and decreased to 1 × 10^−7^ by Polynomial Decay with a power of 0.9. The training was scheduled for a total of 50 epochs. During the fine-tuning, the initial learning rate was set to 5 × 10^−6^ and decreased to 1 × 10^−8^ by Polynomial Decay with a power equal to 1, and the training was scheduled for 20 epochs. For calcification classifications, the same settings were used, except for the scheduled number of epochs during the transfer and fine-tuning, both of which were set to 50. The reported training hyperparameters correspond to the configuration that led to the maximum AUC on the validation set during training.

The loss function measures the extent to which a model’s predictions differ from the desired output, and this deviation needs to be minimized during the training process. In this context, the loss function consists of two components [69,70].

Binary Cross-Entropy (BCE) represents a measure of error in binary classification problems and is based on a heavy penalty for incorrect forecasts. Its formulation is as follows:

(13) $$BCE=-\frac{1}{N}\sum_{i=1}^{N}[y_{i}\log\left(p_{i}\right)+(1-y_{i})\log\left(1-p_{i})\right]$$

where $p_{i}$ is the probability predicted by the model for class 1, $y_{i}$ is the label (0 or 1), and N is the number of examples [71]. During the transfer learning and fine-tuning phases, the Area Under Curve values were tracked, and models were saved only when the AUC improved to prevent overfitting. In this case, the final models are those based on the maximum validation AUC. The models were trained leveraging Google Colab Pro+ resources, Python, Tensorflow, and Keras libraries.

## 3. Results

This section presents the results of the breast lesion classification model based on matRadiomics. The evaluation was performed using stratified k-fold cross-validation with k = 10, and the percentage of data reserved for internal testing was set at 30% (test set). The external validation was performed using the database of the Fondazione G. Giglio Institute in Cefalù. Finally, the results obtained through DL are also presented.

### 3.1. CBIS-DDSM Database

**Figure 1:**
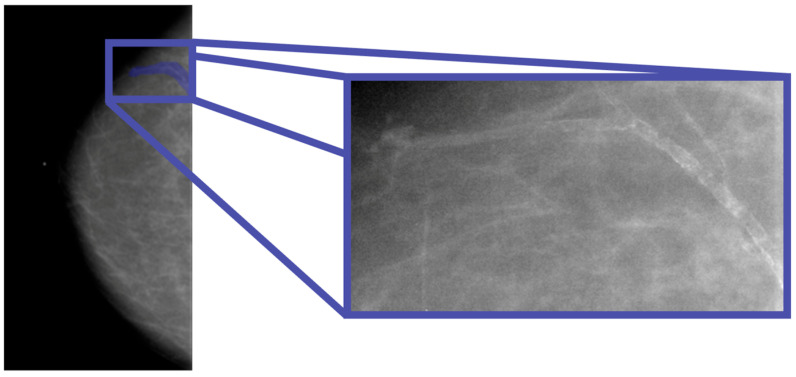
Mammography showing microcalcification within the ROI (Patient P_00038).

**Figure 2:**
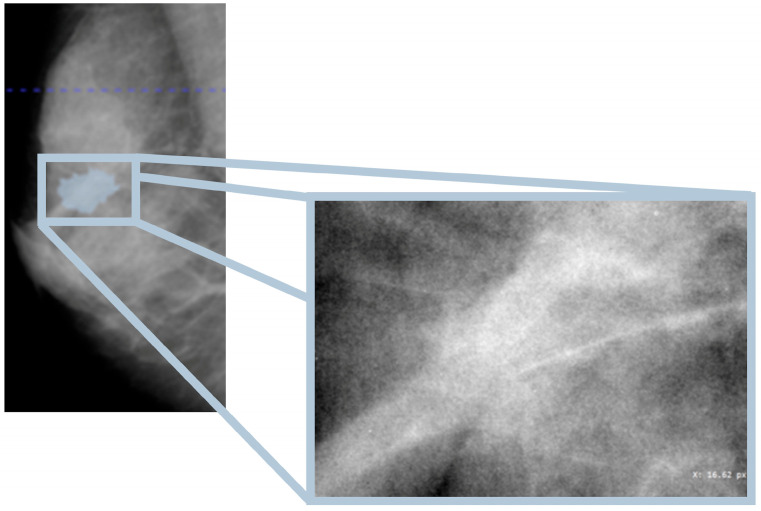
Mammography showing mass within the ROI (Patient P_00612).

In this study, the database used is the CBIS-DDSM, which contains mammographic images representative of the two types of lesions (masses and calcifications) accompanied by appropriate ROI masks, identifying the lesions. In particular, with regard to calcifications, those masks that did not represent the lesion, for example, those that covered the entire breast, were excluded from the study. In Figure 1, an example of a mammographic image containing a microcalcification accompanied by its ROI mask and its cropped image is shown, while in Figure 2, there is a similar example but with an image containing a mass.

### 3.2. Preprocessing Results

**Figure 3:**
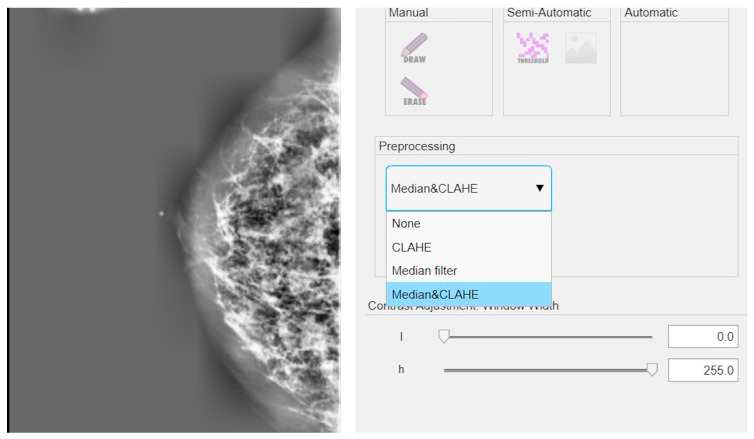
Example of application of CLAHE and median filter in cascade on a mammographic image.

The results discussed in this section are based on the cascade application of the CLAHE method and the median filter. An example of the implementation of these techniques on a mammographic image is shown in Figure 3.

### 3.3. Machine Learning-Based Radiomics

As an initial step, the distinction between masses and calcifications was examined using the entire dataset, which includes 1219 images (638 masses and 581 microcalcifications). Through a PBC analysis, the shape feature original_shape2D_sphericity was identified as a key discriminative factor. This finding is particularly relevant, as it confirms that the model distinguishes lesions based on their shape—masses tend to be more rounded and spherical, whereas calcifications often appear elongated or thread-like.

**Figure 4:**
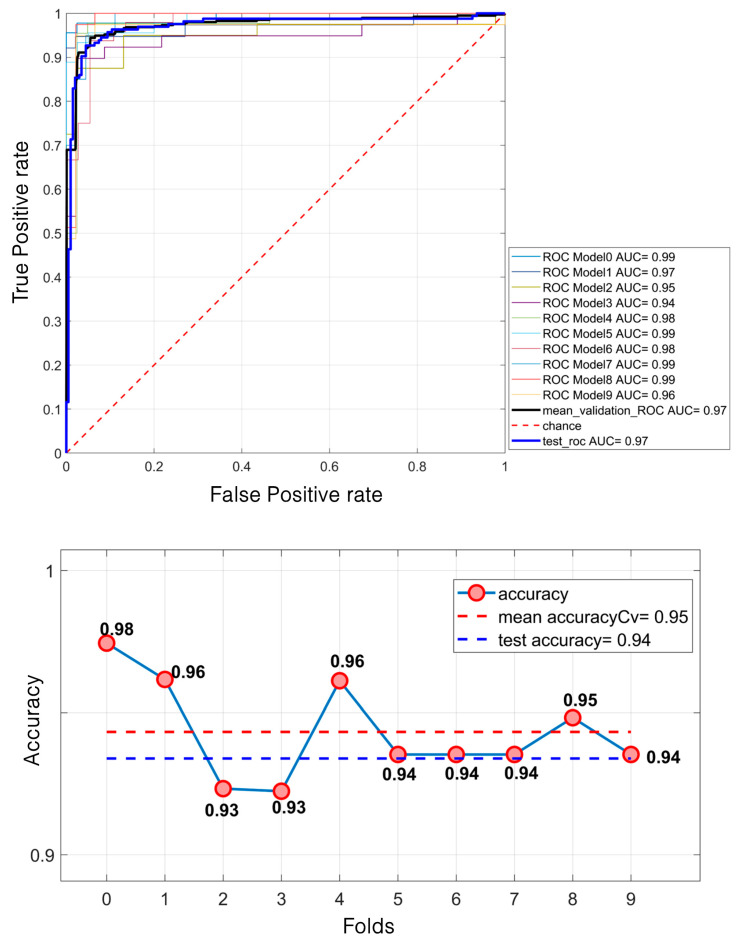
Lesion differentiation task: ROC curve (**up**); accuracies (**down**).

The results presented in Table 1 highlight the strong differentiation capability of the LDA model, while Figure 4 illustrates the corresponding ROC curve (up) and accuracy metrics (down), further validating the model’s performance. Indeed, regarding the differentiation phase, the model achieved high performance in both the validation and test sets. The mean ROC AUC reached 97.42%, with a test ROC AUC of 97.08%. The accuracy was similarly high, with 95% during the validation and 94% on the test set.

In the context of lesion classification, the model is designed to differentiate between benign and malignant cases. Compared to the previous task, the selected features evolve, reflecting the need for different types of information.

For masses, the key selected feature is wavelet_LL_firstorder_Energy, which is no longer related to shape but to texture. This shift indicates that the model requires more in-depth information beyond morphology, as benign and malignant masses often exhibit similar shapes. The texture analysis enables the detection of subtle variations in intensity distribution, thereby enhancing the classification accuracy.

Conversely, for calcifications, the selected feature is original_shape2D_perimeter, emphasizing the role of shape in classification. This suggests that benign and malignant calcifications exhibit more pronounced morphological differences compared to masses, making perimeter a critical distinguishing factor.

**Figure 5:**
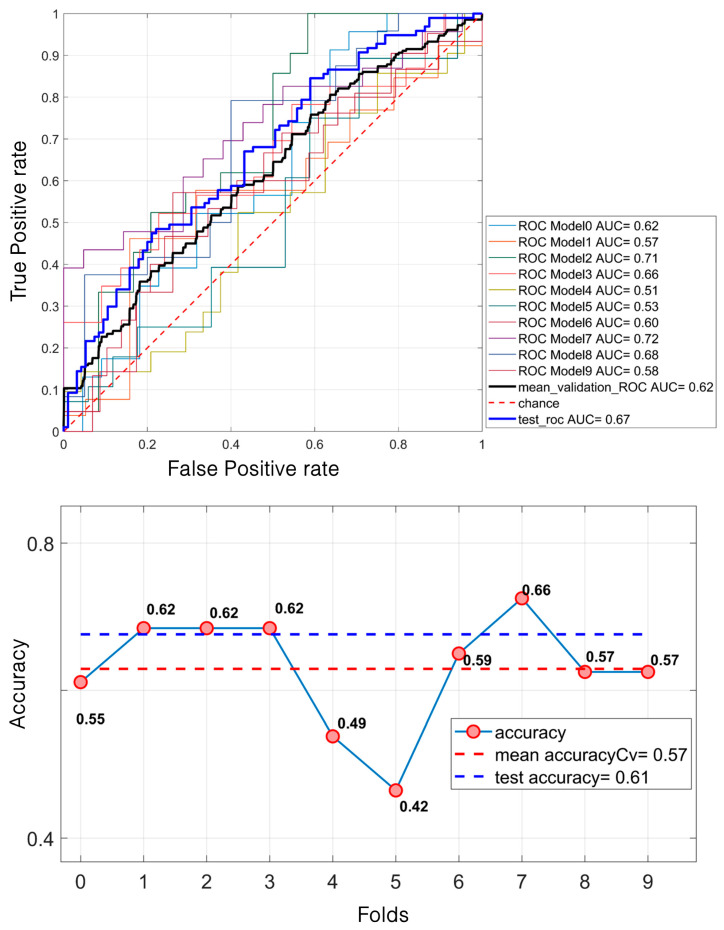
LDA classification of 638 masses. For the ROC curves (**up**), ten different curves are shown, corresponding to the ten folds obtained through the k-fold cross-validation strategy, implemented to ensure greater robustness of the results. The black curve represents the average ROC curve across all folds. Similarly, for each fold, the corresponding accuracy values are reported, with the mean accuracy (**down**) indicated at the bottom of the figure.

Regarding mass classification, the LDA model demonstrated a moderate performance. Specifically, it achieved a mean validation ROC AUC of 61.53%, with a test ROC AUC of 67.14%. The mean accuracy during the validation was 57.43%, increasing to 61% on the test set (Figure 5).

**Figure 6:**
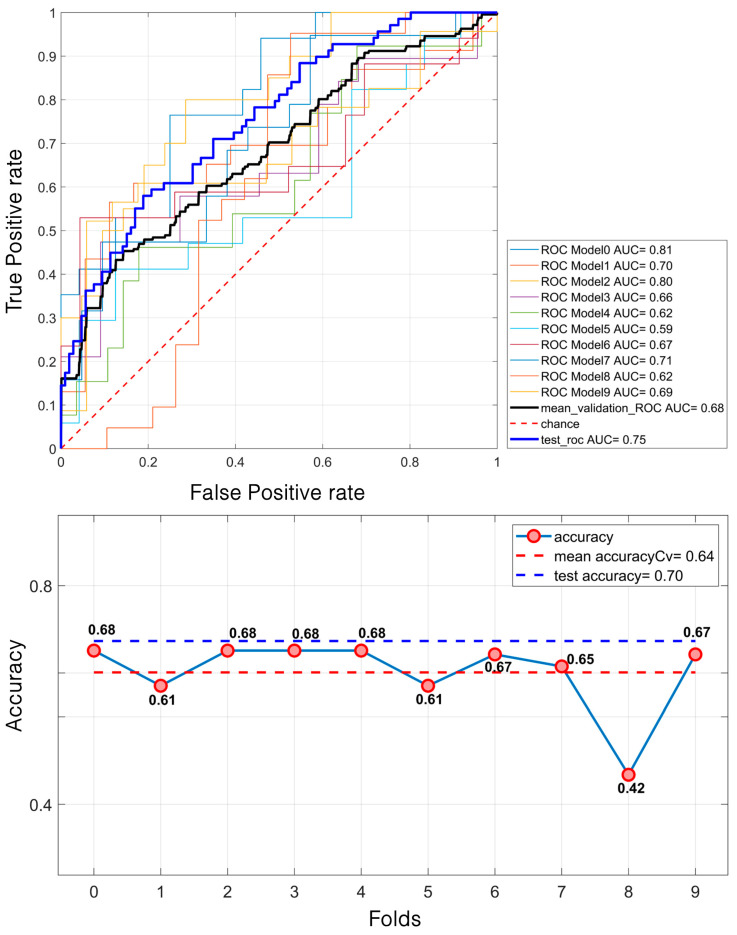
LDA classification of 581 calcifications. For the ROC curves (**up**), ten different curves are shown, corresponding to the ten folds obtained through the k-fold cross-validation strategy, implemented to ensure greater robustness of the results. The black curve represents the average ROC curve across all folds. Similarly, for each fold, the corresponding accuracy (**down**) values are reported, with the mean accuracy indicated at the bottom of the figure.

In contrast, the results for the calcifications were notably higher. The model attained a mean validation ROC AUC of 68.28% and a test ROC AUC of 75%. The accuracy followed a similar trend, with a mean of 64% during the validation and 70% on the test set (Figure 6).

**Figure 7:**
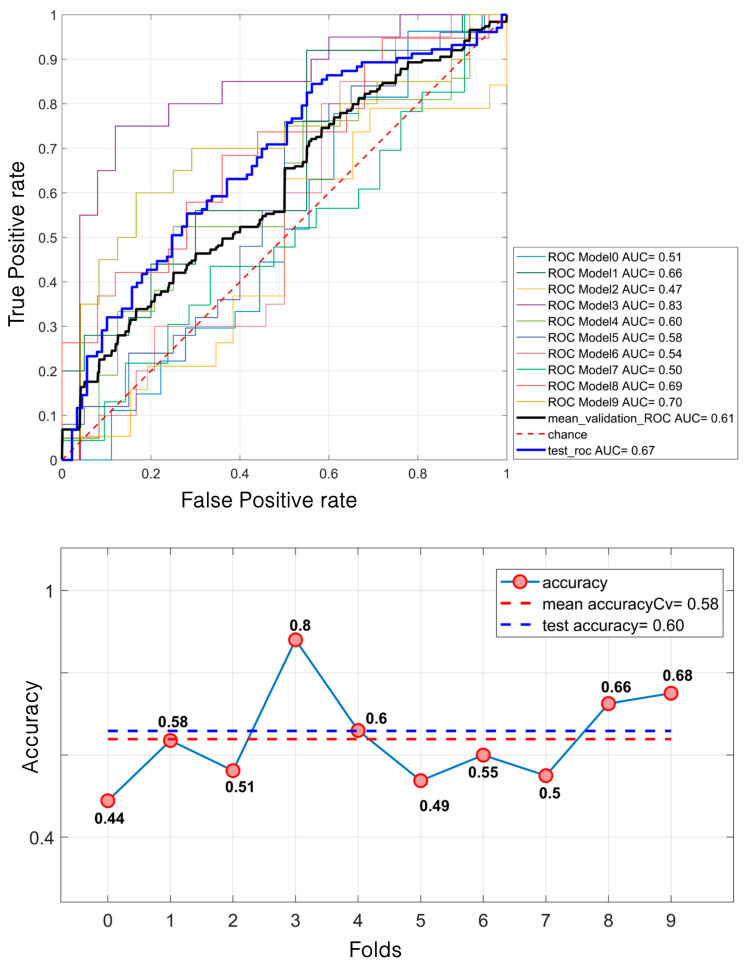
SVM classification of 638 masses. For the ROC curves (**up**), ten different curves are shown, corresponding to the ten folds obtained through the k-fold cross-validation strategy, implemented to ensure greater robustness of the results. The black curve represents the average ROC curve across all folds. Similarly, for each fold, the corresponding accuracy (**down**) values are reported, with the mean accuracy indicated at the bottom of the figure.

**Figure 8:**
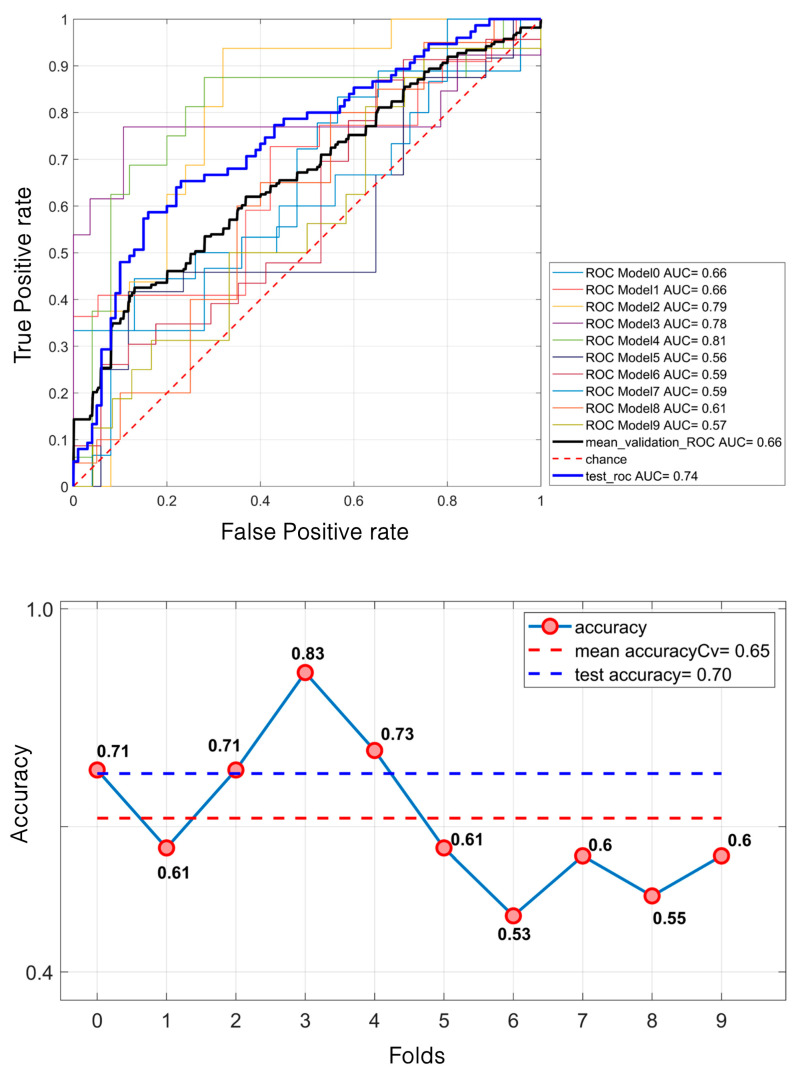
SVM classification of 581 microcalcifications. For the ROC curves (**up**), ten different curves are shown, corresponding to the ten folds obtained through the k-fold cross-validation strategy, implemented to ensure greater robustness of the results. The black curve represents the average ROC curve across all folds. Similarly, for each fold, the corresponding accuracy values are reported, with the mean accuracy (**down**) indicated at the bottom of the figure.

Regarding masses, the SVM model demonstrated a relatively modest performance. It achieved a mean validation ROC AUC of 61% and a test ROC AUC of 66.7%. The mean accuracy during the validation was 58% and 60% for the test set (Figure 7). In contrast, for calcifications, the model performed better. The mean validation ROC AUC reached 66%, with a test ROC AUC of 74.31%. The accuracy also improved, with a mean of 65% during the validation and 70% for the test set (Figure 8).

These results indicate that SVM struggles with mass classification, likely due to the similarity in morphological patterns between benign and malignant lesions. However, calcifications appear to be more effectively distinguished, reinforcing the idea that shape-related features play a crucial role in their classification.

**Figure 9:**
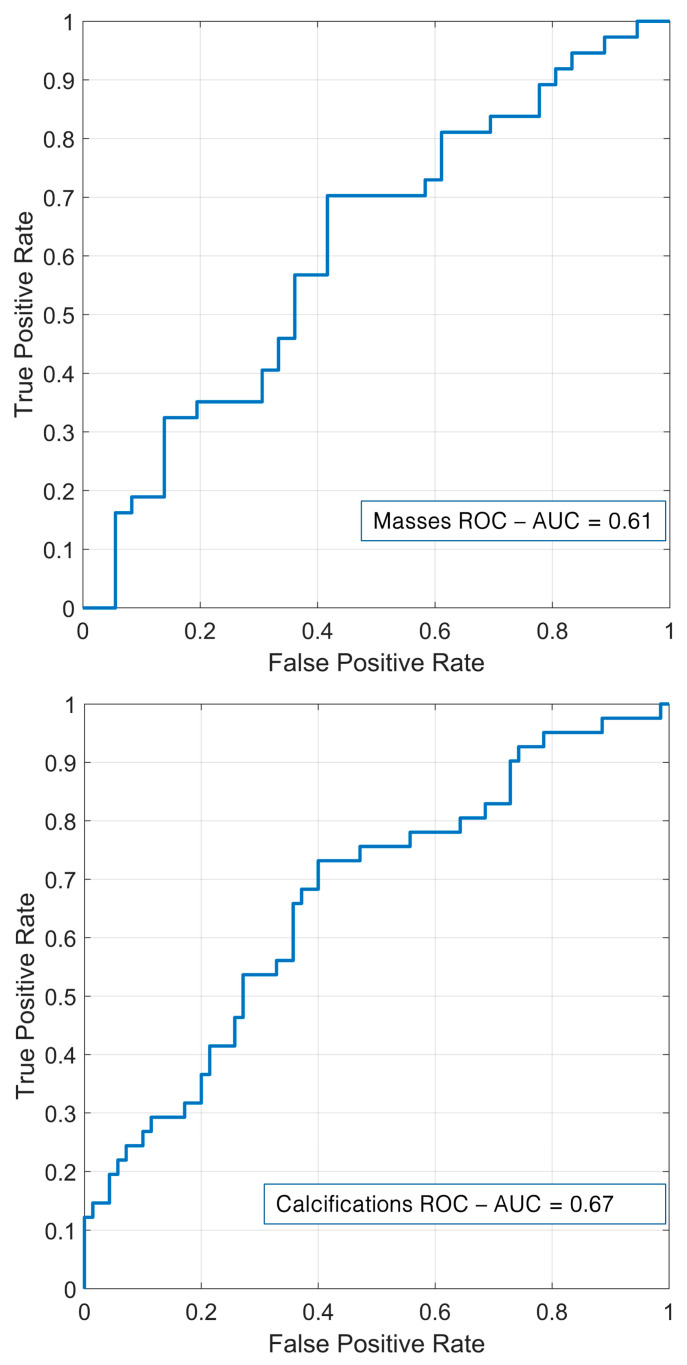
External validation for masses (**up**) and calcifications (**down**).

Based on the LDA, the performance of the external validation based on the private database of the Fondazione G. Giglio Institute of Cefalù is shown in Table 2 and Figure 9. Regarding masses, the model demonstrated a relatively modest performance across all metrics. It achieved an AUC of 61.48% and an accuracy of 63.01%. The recall was 58.33%, and the specificity reached 67.57%, while the precision and F1-score were 63.64% and 60.87%, respectively. In contrast, for calcifications, the performance was consistently higher. The model achieved an AUC of 66.73% and an accuracy of 64.86%. The recall was 60%; the specificity improved to 73.17%, and both the precision and F1-score were notably higher at 79.25% and 68.29%, respectively.

### 3.4. Deep Learning

**Figure 10:**
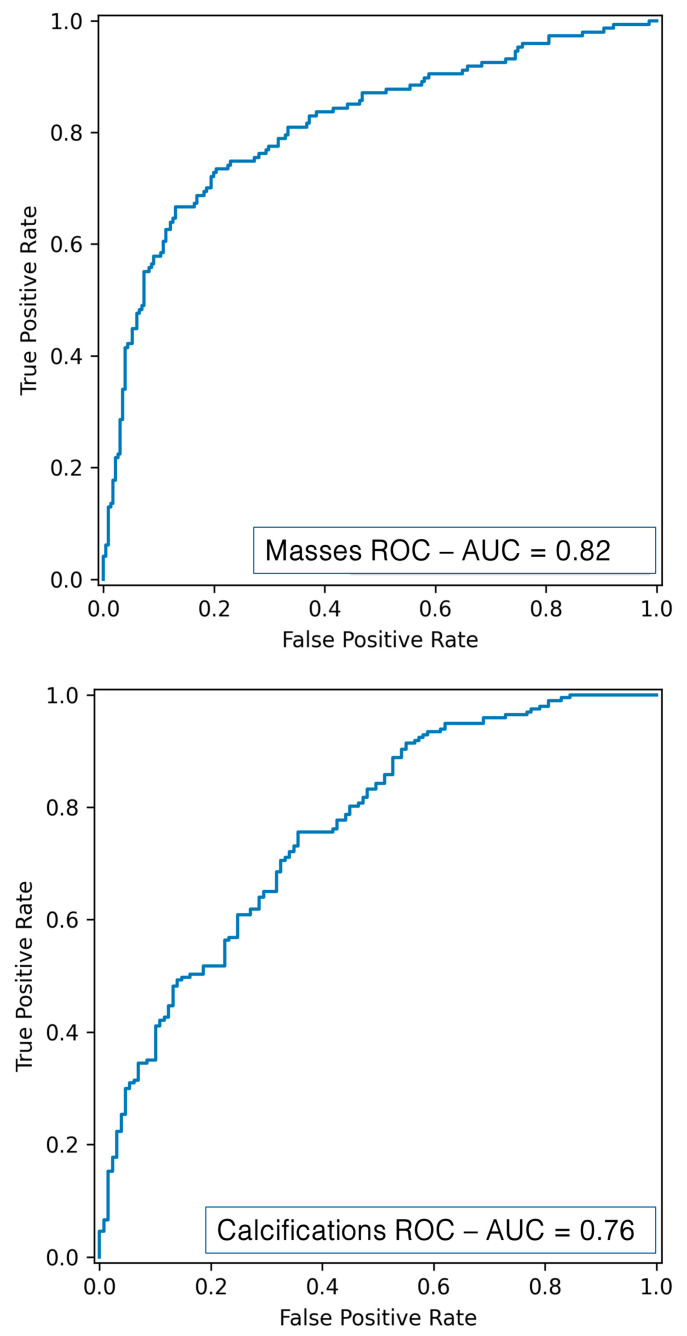
ROC curve for mass (**up**) and calcification (**down**) classification using DL.

Through deep radiomics, performance metrics superior to those obtained with the traditional radiomics flow were obtained, confirming the power of CNN networks that work directly on images. As shown in Table 3 and Figure 10, the DL mass classification resulted in an AUC of 81.52%, an accuracy of 78%, a recall of 66.70%, a precision of 74.24%, and an F1-score of 70.25%, while the DL calcification classification resulted in an AUC of 76.24%, an accuracy of 71.10%, a recall of 85.78%, a precision of 81.96%, and an F1-score of 70.25%.

## 4. Discussion

This study aimed to compare a radiomics workflow based on an ML with a DL approach for the differentiation and classification of breast lesions. Unlike previous studies, ours stands out in the following aspects:

- The integration of a fully automated radiomics workflow based on matRadiomics, which enables end-to-end radiomics processing from image visualization to classification within a unified platform. This integrated solution distinguishes our approach from others that require separate tools for different steps.
- A direct comparison between a radiomics workflow and a DL architecture, focusing on the classification of both masses and microcalcifications—a combination that is not consistently addressed together in the previous literature.
- An external validation to add an important dimension of generalizability, which is often lacking in similar studies.
- Preprocessing techniques (CLAHE and median filtering) were applied to improve image quality [72] by reducing noise and artifacts and enhancing radiomics feature extraction, a step that is often underrepresented in similar comparative studies.

The adoption of radiomics aligns with recent advancements in automated diagnostic tools for medical imaging [73]. The results obtained from ML-based breast lesion classification showed suboptimal performance. The LDA model performed better than SVM with an average validation AUC of 68.28% for microcalcifications and 61.53% for masses. We employed a private dataset, offering an external validation framework to investigate the generalization to independent cohorts. In this case, AUC values of 66.73% and 61.48% were obtained, respectively. We fully acknowledge that the limited size of the external validation dataset could represent a potential risk of overfitting. This dataset was not specifically designed to be balanced with the training set, being collected retrospectively from a private hospital during the time of the study but was used exclusively to test the generalizability of our model. Future studies with larger and more diverse external cohorts will be essential to further validate the model and reduce any residual risk of overfitting. Furthermore, our results were significantly lower than a study based on a different public dataset, in which the authors in [74] reported an AUC of 88% using LDA, with SVM providing slightly worse performance, as in our case. However, a direct comparison is not feasible due to the differences in datasets. Given the limitations observed in our study with traditional ML techniques, a second phase of development was undertaken to explore the potential of DL models. This phase aimed to determine whether advanced neural networks could enhance the classification performance in distinguishing between benign and malignant lesions. In this context, the EfficientNetB6 model outperformed the radiomics approach, demonstrating its potential for improved diagnostic accuracy, with an AUC of 81.52% and 76.24%, respectively. Indeed, EfficientNetB6 inherits the general advantages of DL models over traditional ML models. DL models can learn to extract features tailored for the specific task (in this case, malignant vs. benign discrimination), thus enhancing personalized solutions, while ML models usually rely on pre-determined mathematical features. Moreover, DL models can focus broadly on the entire input image, learning features not only related to the target but also to the target and its relationship with the surrounding tissues. However, DL model training requires a much larger amount of data compared to ML models. To overcome such an issue, transfer learning and fine-tuning can be leveraged. Indeed, the adopted EfficientNetB6 model was loaded with pre-trained weights based on ImageNet datasets. Firstly, the net was adapted to our specific task, adding a new classification head and adopting a fast-training schedule with large learning rates, and then the network was fine-tuned using smaller learning rates. This approach can be used to adapt DL models to different datasets and, of course, EfficientNetB6. Particularly, EfficientNetB6, thanks to its unique compound scaling capability, can optimize its depth, width, and resolution simultaneously based on input. This unique feature is valid across different datasets.

In the literature, Gerbasi et al. [75] achieved remarkable results in breast lesion classification using the same dataset (CBIS-DDSM), obtaining an AUC of 89% through a fully automated pipeline. Specifically, their approach, DeepMiCa, consisted of the following: (1) preprocessing of raw scans, (2) automatic patch-based semantic segmentation using a U-Net-based network, and (3) classification of detected lesions through a deep transfer-learning approach. The most significant difference compared to our work lies in the segmentation strategy. Unlike our study, DeepMiCa performed a new segmentation of the target lesions. Since CBIS-DDSM is a public dataset, many segmentations may contain substantial errors, negatively impacting the classification performance. Future research efforts will be aimed at overcoming this limitation by automating the segmentation process [38,76]. The study shown in [77] proposes an advanced technique for breast cancer classification by combining a CNN with an enhanced metaheuristic optimization algorithm. To overcome existing limitations, the authors modified the Ant Colony Optimization (ACO) algorithm by integrating it with opposition-based learning (OBL), creating the Enhanced Ant Colony Optimization (EACO). This method was used to optimize hyperparameters in the CNN architecture, specifically combining ResNet101 with the EACO algorithm, resulting in the EACO–ResNet101 model. Experimental analyses conducted on the CBIS-DDSM dataset demonstrated that the proposed model achieved an accuracy of 98.63%, outperforming traditional methods. Finally, a hybrid system combining ResNet50 with SVM achieved an AUC of 98.46% on a small subset of 330 cases of the CBIS-DDSM dataset, demonstrating the potential of integrating deep learning with traditional machine learning approaches [78]. These findings underscore the potential of DL methodologies in enhancing diagnostic accuracy for breast cancer detection.

Given these considerations, future developments will focus on enhancing the radiomics pipeline by integrating DL models capable of leveraging raw imaging data directly. In this study, the main goal was not to achieve a state-of-the-art classification performance but, rather, to conduct a comparative evaluation between a traditional ML-based radiomics workflow and a DL approach using a publicly available and widely used dataset. Although other studies have reported higher classification performance on the same dataset, it is important to note that these works have re-segmented the lesions to improve the reliability of the ground truth and reduce the impact of possible errors in the original annotations. In our study, we retained the original segmentations provided in the CBIS-DDSM dataset to evaluate the comparison even in suboptimal conditions. Our results demonstrate that DL models significantly outperform ML-based radiomics models, even when working with potentially noisy or imprecise segmentations. This highlights the added value and resilience of DL methods in such scenarios. Radiomics is used to quantify imaging phenotypes through predefined features, such as texture, shape, and intensity; its performance is highly dependent on segmentation quality and feature selection techniques. Conversely, DL models benefit from end-to-end learning and do not require manual feature engineering. However, future work will focus on improving the segmentation quality, either by re-segmenting lesions manually or by incorporating advanced automatic segmentation techniques. These efforts will be crucial for enhancing the classification performance. In addition, the use of EfficientNetB6 in forthcoming studies aims to explore the synergy between radiomics and neural networks, potentially improving the classification accuracy while preserving the interpretability of handcrafted features. Indeed, the advantage of radiomics features is their clear interpretability since they are based on explainable mathematical expressions. Conversely, DL models are usually described as black boxes, and the extracted features could not reflect the clinical and physical properties of the object investigated. However, recent developments in explainable artificial intelligence (XAI) tried to address this issue by leveraging visual explanation techniques such as GradCam [79], GradCam++ [80], and Saliency maps. These techniques highlight through heatmaps the parts of the input image that contributed the most to the final prediction. For example, leveraging GradCam++, it is possible to see the evolution of the extracted features after each convolutional block from earlier layers to deeper layers, and it emerges that features extracted at earlier layers are sharper and highlight edges, corners, and contours, while they become more abstract at deeper layers. Therefore, XAI can be used to enhance model interpretability and will be incorporated in future works to understand which part of a calcification and/or mass contributes the most to the discrimination between benign and malignant subtypes. Indeed, a GradCam++ heatmap can be used to re-segment the calcification and/or mass and divide it into zones to generate habitats, from where radiomics features can be extracted and DL models could be retrained. Moreover, incorporating image rotation and multi-view image fusion could further optimize lesion characterization. Furthermore, DL models can be particularly sensitive to class imbalance, which may introduce bias and affect model generalizability. In the present study, we intentionally chose not to apply specific data balancing techniques to maintain methodological consistency with the ML workflow, where no balancing was applied either. Future work will include dedicated investigations into how data balancing strategies may influence model performance. Beyond preprocessing improvements, the adoption of ensemble learning techniques and more sophisticated feature selection strategies could significantly enhance classification robustness and reliability. The growing trend of hybrid models, which combine traditional ML architectures with DL, seems to enable greater diagnostic accuracy and clinical integration [81,82,83]. For example, combining radiomics-based feature selection with concatenated neural networks (CNNs) for final classification allows the model to leverage both manually created and machine-learned features. Ultimately, while radiomics remains a powerful tool for lesion characterization, its full potential can be unlocked by bridging the gap between feature engineering and data-driven DL approaches. By refining the proposed workflow and incorporating state-of-the-art AI techniques, this research lays the groundwork for more accurate, reliable, and clinically applicable tools in breast cancer detection.

## 5. Conclusions

The proposed study highlights the significant potential of artificial intelligence models for breast lesion classification. Traditional models, such as LDA and SVM, have shown promising results. The use of a proprietary dataset has strengthened the model’s generalizability, ensuring its clinical applicability. However, DL approaches promise to further improve the classification accuracy and robustness, providing an exciting direction for future research with the potential to significantly enhance diagnostic tools in clinical settings.

## Figures and Tables

**Table 1:** Results of discrimination between masses and calcifications in the internal test set.

ROC AUC	Test ROC AUC	Accuracy	Test Accuracy
97.42%	97.08%	95%	94%

**Table 2:** Machine learning performance on the external test set.

Classification Type	AUC	Accuracy	Recall	Specificity	Precision	F1-Score
Masses	61.48%	63.01%	58.33%	67.57%	63.64%	60.87%
Calcifications	66.73%	64.86%	60%	73.17%	79.25%	68.29%

**Table 3:** Results obtained using deep radiomics for mass and calcification classifications for the internal test set.

Classification Type	AUC	Accuracy	Recall	Specificity	Precision	F1-Score
Masses	81.52%	78%	66.70%	85.28%%	75.39%	70.25%
Calcifications	76.24%	71.1%	85.78%	48.84%	81.96%	78.24%

## Data Availability

The original contributions presented in this study are included in the article. Further inquiries can be directed to the corresponding author.

## References

[1] Benson J.R., Jatoi I., Keisch M., Esteva F.J., Makris A., Jordan V.C. (2009). Early Breast Cancer. Lancet.

[2] Carlson R.W., Allred D.C., Anderson B.O., Burstein H.J., Carter W.B., Edge S.B., Erban J.K., Farrar W.B., Forero A., Giordano S.H. (2011). Invasive Breast Cancer. J. Natl. Compr. Cancer Netw..

[3] Tan P.H., Ellis I., Allison K., Brogi E., Fox S.B., Lakhani S., Lazar A.J., Morris E.A., Sahin A., Salgado R. (2020). The 2019 World Health Organization Classification of Tumours of the Breast. Histopathology.

[4] Muller K., Jorns J.M., Tozbikian G. (2022). What’s New in Breast Pathology 2022: WHO 5th Edition and Biomarker Updates. J. Pathol. Transl. Med..

[5] Kim S., Tran T.X.M., Song H., Park B. (2022). Microcalcifications, Mammographic Breast Density, and Risk of Breast Cancer: A Cohort Study. Breast Cancer Res..

[6] Calisto F.M., Nunes N., Nascimento J.C. BreastScreening. Proceedings of the International Conference on Advanced Visual Interfaces.

[7] Sternlicht M.D. (2005). Key Stages in Mammary Gland Development: The Cues That Regulate Ductal Branching Morphogenesis. Breast Cancer Res..

[8] Biswas S.K., Banerjee S., Baker G.W., Kuo C.-Y., Chowdhury I. (2022). The Mammary Gland: Basic Structure and Molecular Signaling during Development. Int. J. Mol. Sci..

[9] Gao Y., Moy L., Heller S.L. (2021). Digital Breast Tomosynthesis: Update on Technology, Evidence, and Clinical Practice. RadioGraphics.

[10] Richman I.B., Long J.B., Hoag J.R., Upneja A., Hooley R., Xu X., Kunst N., Aminawung J.A., Kyanko K.A., Busch S.H. (2021). Comparative Effectiveness of Digital Breast Tomosynthesis for Breast Cancer Screening Among Women 40–64 Years Old. JNCI J. Natl. Cancer Inst..

[11] Wang Y., Li Y., Song Y., Chen C., Wang Z., Li L., Liu M., Liu G., Xu Y., Zhou Y. (2022). Comparison of Ultrasound and Mammography for Early Diagnosis of Breast Cancer among Chinese Women with Suspected Breast Lesions: A Prospective Trial. Thorac. Cancer.

[12] Lee S.E., Yoon J.H., Son N.-H., Han K., Moon H.J. (2024). Screening in Patients With Dense Breasts: Comparison of Mammography, Artificial Intelligence, and Supplementary Ultrasound. Am. J. Roentgenol..

[13] Chang A.Y., Joe B.N. (2020). Breast MRI Finds More Invasive Cancers than Digital Breast Tomosynthesis in Women with Dense Breasts Undergoing Screening. Radiol. Imaging Cancer.

[14] Bancroft A., Santa Cruz J., Levett K., Nguyen Q.D. (2024). Incidental Breast Hemangioma on Breast MRI: A Case Report. Cureus.

[15] Magny S.J., Shikhman R., Keppke A.L. (2023). Breast Imaging Reporting and Data System.

[16] Esposito D., Paternò G., Ricciardi R., Sarno A., Russo P., Mettivier G. (2024). A Pre-Processing Tool to Increase Performance of Deep Learning-Based CAD in Digital Breast Tomosynthesis. Health Technol..

[17] Balma M., Liberini V., Buschiazzo A., Racca M., Rizzo A., Nicolotti D.G., Laudicella R., Quartuccio N., Longo M., Perlo G. (2023). The Role of Theragnostics in Breast Cancer: A Systematic Review of the Last 12 Years. Curr. Med. Imaging.

[18] Laudicella R., Comelli A., Liberini V., Vento A., Stefano A., Spataro A., Crocè L., Baldari S., Bambaci M., Deandreis D. (2022). [^68^Ga]DOTATOC PET/CT Radiomics to Predict the Response in GEP-NETs Undergoing [^177^Lu]DOTATOC PRRT: The Theragnomics Concept. Cancers.

[19] Perniciano A., Loddo A., Di Ruberto C., Pes B. (2024). Insights into Radiomics: Impact of Feature Selection and Classification. Multimed. Tools Appl..

[20] Marinov S., Buliev I., Cockmartin L., Bosmans H., Bliznakov Z., Mettivier G., Russo P., Bliznakova K. (2021). Radiomics Software for Breast Imaging Optimization and Simulation Studies. Phys. Medica.

[21] Najjar R. (2023). Redefining Radiology: A Review of Artificial Intelligence Integration in Medical Imaging. Diagnostics.

[22] Jha A.K., Mithun S., Sherkhane U.B., Dwivedi P., Puts S., Osong B., Traverso A., Purandare N., Wee L., Rangarajan V. (2023). Emerging Role of Quantitative Imaging (Radiomics) and Artificial Intelligence in Precision Oncology. Explor. Target. Antitumor Ther..

[23] Zhang W., Guo Y., Jin Q. (2023). Radiomics and Its Feature Selection: A Review. Symmetry.

[24] Vial A., Stirling D., Field M., Ros M., Ritz C., Carolan M., Holloway L., Miller A.A. (2018). The Role of Deep Learning and Radiomic Feature Extraction in Cancer-Specific Predictive Modelling: A Review. Transl. Cancer Res..

[25] Wu N., Phang J., Park J., Shen Y., Huang Z., Zorin M., Jastrzebski S., Fevry T., Katsnelson J., Kim E. (2020). Deep Neural Networks Improve Radiologists’ Performance in Breast Cancer Screening. IEEE Trans. Med. Imaging.

[26] Chassagnon G., Vakalopoulou M., Régent A., Zacharaki E.I., Aviram G., Martin C., Marini R., Bus N., Jerjir N., Mekinian A. (2020). Deep Learning–Based Approach for Automated Assessment of Interstitial Lung Disease in Systemic Sclerosis on CT Images. Radiol. Artif. Intell..

[27] Avanzo M., Wei L., Stancanello J., Vallières M., Rao A., Morin O., Mattonen S.A., El Naqa I. (2020). Machine and Deep Learning Methods for Radiomics. Med. Phys..

[28] Avanzo M., Porzio M., Lorenzon L., Milan L., Sghedoni R., Russo G., Massafra R., Fanizzi A., Barucci A., Ardu V. (2021). Artificial Intelligence Applications in Medical Imaging: A Review of the Medical Physics Research in Italy. Phys. Medica.

[29] Carriero A., Groenhoff L., Vologina E., Basile P., Albera M. (2024). Deep Learning in Breast Cancer Imaging: State of the Art and Recent Advancements in Early 2024. Diagnostics.

[30] Ferro A., Bottosso M., Dieci M.V., Scagliori E., Miglietta F., Aldegheri V., Bonanno L., Caumo F., Guarneri V., Griguolo G. (2024). Clinical Applications of Radiomics and Deep Learning in Breast and Lung Cancer: A Narrative Literature Review on Current Evidence and Future Perspectives. Crit. Rev. Oncol./Hematol..

[31] Lotter W., Diab A.R., Haslam B., Kim J.G., Grisot G., Wu E., Wu K., Onieva J.O., Boyer Y., Boxerman J.L. (2021). Robust Breast Cancer Detection in Mammography and Digital Breast Tomosynthesis Using an Annotation-Efficient Deep Learning Approach. Nat. Med..

[32] Niu J., Li H., Zhang C., Li D. (2021). Multi-Scale Attention-Based Convolutional Neural Network for Classification of Breast Masses in Mammograms. Med. Phys..

[33] Tan M., Le Q.V. EfficientNet: Rethinking Model Scaling for Convolutional Neural Networks. Proceedings of the 36th International Conference on Machine Learning (ICML).

[34] He K., Zhang X., Ren S., Sun J. Deep Residual Learning for Image Recognition. Proceedings of the IEEE Conference on Computer Vision and Pattern Recognition.

[35] Stefano A., Bini F., Lauciello N., Pasini G., Marinozzi F., Russo G. (2024). Implementation of Automatic Segmentation Framework as Preprocessing Step for Radiomics Analysis of Lung Anatomical Districts. BioMedInformatics.

[36] Huang G., Liu Z., Van Der Maaten L., Weinberger K.Q. Densely Connected Convolutional Networks. Proceedings of the 30th IEEE Conference on Computer Vision and Pattern Recognition (CVPR).

[37] Szegedy C., Liu W., Jia Y., Sermanet P., Reed S., Anguelov D., Erhan D., Vanhoucke V., Rabinovich A. Going Deeper with Convolutions. Proceedings of the IEEE Computer Society Conference on Computer Vision and Pattern Recognition.

[38] Kumar Saha D., Hossain T., Safran M., Alfarhood S., Mridha M.F., Che D. (2024). Segmentation for Mammography Classification Utilizing Deep Convolutional Neural Network. BMC Med. Imaging.

[39] Alghamdi S.S. (2023). The Application of Artificial Intelligence in Detecting Breast Lesions with Medical Imaging: A Literature Review. Int. J. Biomed..

[40] Bini F., Missori E., Pucci G., Pasini G., Marinozzi F., Forte G.I., Russo G., Stefano A. (2024). Preclinical Implementation of MatRadiomics: A Case Study for Early Malformation Prediction in Zebrafish Model. J. Imaging.

[41] Pasini G., Bini F., Russo G., Comelli A., Marinozzi F., Stefano A. (2022). MatRadiomics: A Novel and Complete Radiomics Framework, from Image Visualization to Predictive Model. J. Imaging.

[42] Sawyer-Lee R., Francisco G., Assaf H., Daniel R. (2016). Curated Breast Imaging Subset of Digital Database for Screening Mammography (CBIS-DDSM). DataCite Commons.

[43] Stefano A. (2024). Challenges and Limitations in Applying Radiomics to PET Imaging: Possible Opportunities and Avenues for Research. Comput. Biol. Med..

[44] (2020). Pyradiomics Documentation Release v3.0.Post5+gf06ac1d Pyradiomics Community. https://pyradiomics.readthedocs.io/en/v3.0/.

[45] Horng H., Singh A., Yousefi B., Cohen E.A., Haghighi B., Katz S., Noël P.B., Shinohara R.T., Kontos D. (2022). Generalized ComBat Harmonization Methods for Radiomic Features with Multi-Modal Distributions and Multiple Batch Effects. Sci. Rep..

[46] Bauckneht M., Pasini G., Di Raimondo T., Russo G., Raffa S., Donegani M.I., Dubois D., Peñuela L., Sofia L., Celesti G. (2025). [^18^F]PSMA-1007 PET/CT-Based Radiomics May Help Enhance the Interpretation of Bone Focal Uptakes in Hormone-Sensitive Prostate Cancer Patients. Eur. J. Nucl. Med. Mol. Imaging.

[47] Pasini G., Russo G., Mantarro C., Bini F., Richiusa S., Morgante L., Comelli A., Russo G.I., Sabini M.G., Cosentino S. (2023). A Critical Analysis of the Robustness of Radiomics to Variations in Segmentation Methods in ^18^F-PSMA-1007 PET Images of Patients Affected by Prostate Cancer. Diagnostics.

[48] Pasini G., Stefano A., Russo G., Comelli A., Marinozzi F., Bini F. (2023). Phenotyping the Histopathological Subtypes of Non-Small-Cell Lung Carcinoma: How Beneficial Is Radiomics?. Diagnostics.

[49] Vernuccio F., Arnone F., Cannella R., Verro B., Comelli A., Agnello F., Stefano A., Gargano R., Rodolico V., Salvaggio G. (2021). Diagnostic Performance of Qualitative and Radiomics Approach to Parotid Gland Tumors: Which Is the Added Benefit of Texture Analysis?. Br. J. Radiol..

[50] Sukassini M.P., Velmurugan T. Noise Removal Using Morphology and Median Filter Methods in Mammogram Images. Proceedings of the 3rd International Conference on Small & Medium Business.

[51] Nguyen T.P.H., Cai Z., Nguyen K., Keth S., Shen N., Park M. (2020). Pre-Processing Image Using Brightening, CLAHE and RETINEX. Electrical Engineering and Systems Science > Image and Video Processing. arXiv.

[52] Erwin, Ningsih D.R. (2020). Improving Retinal Image Quality Using the Contrast Stretching, Histogram Equalization, and CLAHE Methods with Median Filters. Int. J. Image Graph. Signal Process..

[53] Gonzalez R.C. (2009). Digital Image Processing.

[54] Teng X., Wang Y., Nicol A.J., Ching J.C.F., Wong E.K.Y., Lam K.T.C., Zhang J., Lee S.W.-Y., Cai J. (2024). Enhancing the Clinical Utility of Radiomics: Addressing the Challenges of Repeatability and Reproducibility in CT and MRI. Diagnostics.

[55] Mayerhoefer M.E., Materka A., Langs G., Häggström I., Szczypiński P., Gibbs P., Cook G. (2020). Introduction to Radiomics. J. Nucl. Med..

[56] Corso R., Stefano A., Salvaggio G., Comelli A. (2024). Shearlet Transform Applied to a Prostate Cancer Radiomics Analysis on MR Images. Mathematics.

[57] van Griethuysen J.J.M., Fedorov A., Parmar C., Hosny A., Aucoin N., Narayan V., Beets-Tan R.G.H., Fillion-Robin J.-C., Pieper S., Aerts H.J.W.L. (2017). Computational Radiomics System to Decode the Radiographic Phenotype. Cancer Res..

[58] Rajpoot C.S., Sharma G., Gupta P., Dadheech P., Yahya U., Aneja N. (2024). Feature Selection-Based Machine Learning Comparative Analysis for Predicting Breast Cancer. Appl. Artif. Intell..

[59] Antunes A.R., Matos M.A., Costa L.A., Rocha A.M.A.C., Braga A.C. Feature Selection Optimization for Breast Cancer Diagnosis. Proceedings of the Optimization, Learning Algorithms and Applications.

[60] Matharaarachchi S., Domaratzki M., Muthukumarana S. (2021). Assessing Feature Selection Method Performance with Class Imbalance Data. Mach. Learn. Appl..

[61] Barone S., Cannella R., Comelli A., Pellegrino A., Salvaggio G., Stefano A., Vernuccio F. (2021). Hybrid Descriptive-Inferential Method for Key Feature Selection in Prostate Cancer Radiomics. Appl. Stoch. Models Bus. Ind..

[62] Molina L.C., Belanche L., Nebot A. Feature Selection Algorithms: A Survey and Experimental Evaluation. Proceedings of the 2002 IEEE International Conference on Data Mining.

[63] Guyon I., Elisseeff A. (2003). An Introduction of Variable and Feature Selection. J. Mach. Learn. Res..

[64] Adebiyi M.O., Arowolo M.O., Mshelia M.D., Olugbara O.O. (2022). A Linear Discriminant Analysis and Classification Model for Breast Cancer Diagnosis. Appl. Sci..

[65] Egwom O.J., Hassan M., Tanimu J.J., Hamada M., Ogar O.M. (2022). An LDA–SVM Machine Learning Model for Breast Cancer Classification. BioMedInformatics.

[66] Carrington A.M., Manuel D.G., Fieguth P.W., Ramsay T., Osmani V., Wernly B., Bennett C., Hawken S., Magwood O., Sheikh Y. (2023). Deep ROC Analysis and AUC as Balanced Average Accuracy, for Improved Classifier Selection, Audit and Explanation. IEEE Trans. Pattern Anal. Mach. Intell..

[67] Fawcett T. (2006). An Introduction to ROC Analysis. Pattern Recognit. Lett..

[68] Hajian-Tilaki K. (2013). Receiver Operating Characteristic (ROC) Curve Analysis for Medical Diagnostic Test Evaluation. Casp. J. Intern. Med..

[69] Ding Y. The Impact of Learning Rate Decay and Periodical Learning Rate Restart on Artificial Neural Network. Proceedings of the 2021 2nd International Conference on Artificial Intelligence in Electronics Engineering.

[70] Smaida M., Yaroshchak S., Sasi A.Y.B. (2021). Learning Rate Optimization in CNN for Accurate Ophthalmic Classification. Int. J. Innov. Technol. Explor. Eng. (IJITEE).

[71] Zadeh S.G., Schmid M. (2021). Bias in Cross-Entropy-Based Training of Deep Survival Networks. IEEE Trans. Pattern Anal. Mach. Intell..

[72] Elahi R., Nazari M. (2024). An Updated Overview of Radiomics-Based Artificial Intelligence (AI) Methods in Breast Cancer Screening and Diagnosis. Radiol. Phys. Technol..

[73] Sierra-Franco C.A., Hurtado J., de A. Thomaz V., da Cruz L.C., Silva S.V., Silva-Calpa G.F.M., Raposo A. (2024). Towards Automated Semantic Segmentation in Mammography Images for Enhanced Clinical Applications. J. Imaging Inform. Med..

[74] Fusco R., Piccirillo A., Sansone M., Granata V., Rubulotta M.R., Petrosino T., Barretta M.L., Vallone P., Di Giacomo R., Esposito E. (2021). Radiomics and Artificial Intelligence Analysis with Textural Metrics Extracted by Contrast-Enhanced Mammography in the Breast Lesions Classification. Diagnostics.

[75] Gerbasi A., Clementi G., Corsi F., Albasini S., Malovini A., Quaglini S., Bellazzi R. (2023). DeepMiCa: Automatic Segmentation and Classification of Breast MIcroCAlcifications from Mammograms. Comput. Methods Programs Biomed..

[76] Stefano A., Vitabile S., Russo G., Ippolito M., Marletta F., D’Arrigo C., D’Urso D., Gambino O., Pirrone R., Ardizzone E. (2016). A Fully Automatic Method for Biological Target Volume Segmentation of Brain Metastases. Int. J. Imaging Syst. Technol..

[77] Thirumalaisamy S., Thangavilou K., Rajadurai H., Saidani O., Alturki N., Mathivanan S.K., Jayagopal P., Gochhait S. (2023). Breast Cancer Classification Using Synthesized Deep Learning Model with Metaheuristic Optimization Algorithm. Diagnostics.

[78] Salama W.M., Elbagoury A.M., Aly M.H. (2020). Novel Breast Cancer Classification Framework Based on Deep Learning. IET Image Process..

[79] Selvaraju R.R., Cogswell M., Das A., Vedantam R., Parikh D., Batra D. (2016). Grad-CAM: Visual Explanations from Deep Networks via Gradient-Based Localization. Int. J. Comput. Vis..

[80] Chattopadhyay A., Sarkar A., Howlader P., Balasubramanian V.N. Grad-CAM++: Generalized Gradient-Based Visual Explanations for Deep Convolutional Networks. Proceedings of the 2018 IEEE Winter Conference on Applications of Computer Vision.

[81] Caii W., Wu X., Guo K., Chen Y., Shi Y., Chen J. (2024). Integration of Deep Learning and Habitat Radiomics for Predicting the Response to Immunotherapy in NSCLC Patients. Cancer Immunol. Immunother..

[82] Zhang X., Zhang Y., Zhang G., Qiu X., Tan W., Yin X., Liao L. (2022). Deep Learning With Radiomics for Disease Diagnosis and Treatment: Challenges and Potential. Front. Oncol..

[83] Kim S., Lim J.H., Kim C.H., Roh J., You S., Choi J.S., Lim J.H., Kim L., Chang J.W., Park D. (2024). Deep Learning–Radiomics Integrated Noninvasive Detection of Epidermal Growth Factor Receptor Mutations in Non-Small Cell Lung Cancer Patients. Sci. Rep..

